# A Doctor’s Rough Experience with the “Law”

**Published:** 1887-04

**Authors:** R. G. Williams

**Affiliations:** Whitney, Texas


### A DOCTOR'S ROUGH EXPERIENCE WITH THE "LAW."

*By R. G. Williams, M. D., Whitney, Texas.*

For Daniel's Texas Medical Journal.

IN the summer of 1882 I was summoned to accompany the magistrate and a jury a distance of five miles in the country, to examine a woman who it was supposed had but a few days previous given birth to an infant. I refused to go, and was then notified by the magistrate if I persisted in my refusal to go he would fine me $25 for contempt. I decided to go.

The history of this case is as follows:

A married woman with her husband had living with them an unmarried sister, about 24 years of age. These three were strangers in our section, and for about ten days occupied an old log cabin in a field. They disappeared as unexpectedly as they came. The day after departure some boys, while out hunting, found, a short distance from this old cabin, a dead baby, hidden beneath a pile of corn-stalks. These stalks had been fired, but being rather green had but partially burned. The news of the find soon spread, and at once the magistrate, with his jury, repaired to the designated place, where, too true, the body of a dead infant was found. The body was brought to town, and properly viewed, (no M. D. asked to examine and tell if the baby was born dead or alive) and decently buried.

The next effort made was to find the defaulting family. They were located about five miles away. We, that is, the magistrate, and jury, myself, and about one dozen curiosity seekers, were soon face to face with the supposed infant murderer. I was requested, I prefer to say *ordered*, by the magistrate to make every effort in my power to discover if either woman had quite recently given birth to a child. The crowd retired, leaving me with the two women.

I told them they were under arrest for the murder of an infant, and as a physician, I would advise that the one suspected, and who all along had remained in bed under cover, should permit me to make a vaginal examination. This was pointedly refused. However, after much parleying, I was permitted to examine her breasts. These were full and well distended, and I saw sufficient evidence for me to state she had recently given birth to a child, but that if she would permit a vaginal examination, I might decide to the contrary. She finally gave her consent, and I found a large uterus, a slightly dilated cervix, a large patulous os, and a free lochial discharge. I at once told her she had recently, within the past five days, given birth to a child. She admitted to having had a baby two years previous. Finding she could not deceive me, she said it was true, she had given birth to a baby, and had placed it where found. She then asked me what the law would do with her. I answered I had nothing to do with the law, but as there was a lawyer in the crowd outside, I would send him in. This lawyer, though by no means smart, had shrewdness sufficient to advise her to say the baby was born dead, and that she burried it where found.

As we have no law compelling certain burial forms, this woman was acquitted.

The ride of five miles, two or three hundred words, and finally an examination, consumed the time between the hours of 2 p. m. and 8 p. m. I made out the paltry bill of $5.00 and had it duly presented to that august body, the County Commissioners, who, after an hour's debate upon the subject, decided I was not entitled to any fee.

AGAIN.

On the 21st of this month, March, 1887, I was summoned by a constable to appear before the grand jury at an early hour on the morning of the 28th inst. I answered I would not go; he said if I did not go I would certainly be fined, as the district judge had informed the grand jury that if any one dared to disobey their summons, to report, and he would let them feel the weight of the law. So I no longer hesitated. Early on the morning of the 28th I could have been seen, hurriedly driving for the capitol of our county, 14 miles away, yet at 10 a. m. I reported to the officer of the day, that I was ready and willing to tell the grand jury my every and innermost thoughts. He answered, "they would not assemble until 1 p. m."

At 1 p. m. I was at the door of the grand jury room, where I, with many others, were patiently awaiting our turn, when at 3:40 a member of the grand jury shoved the crown of his head out of the door, saying: "Dr. Williams, we have decided you know nothing that will benefit us, so now you can return home.

After many thanks and well wishes for their future happiness, I drove hastily home, reaching my own fig tree about 6 p. m., to find that at 9 a. m., I had received a call to an obstetric case; at 12:30 p. m., had a call to a child in town, and at 3 p. m., a call two miles out in country.

This was not a very pleasant reflection to me, to know I had ridden 28 miles; had remained for three hours near the door of the assembled jurymen; to finally be told I was not needed; to pay hotel and stable bill, and to know I had lost $14 by enforced absence.

